# Comparative Bacterial Proteomics: Analysis of the Core Genome Concept

**DOI:** 10.1371/journal.pone.0001542

**Published:** 2008-02-06

**Authors:** Stephen J. Callister, Lee Ann McCue, Joshua E. Turse, Matthew E. Monroe, Kenneth J. Auberry, Richard D. Smith, Joshua N. Adkins, Mary S. Lipton

**Affiliations:** 1 Biological Sciences Division, Pacific Northwest National Laboratory, Richland, Washington, United States of America; 2 Computational Sciences and Mathematics Division, Pacific Northwest National Laboratory, Richland, Washington, United States of America; 3 Environmental Molecular Sciences Laboratory, Pacific Northwest National Laboratory, Richland, Washington, United States of America; University of Queensland, Australia

## Abstract

While comparative bacterial genomic studies commonly predict a set of genes indicative of common ancestry, experimental validation of the existence of this core genome requires extensive measurement and is typically not undertaken. Enabled by an extensive proteome database developed over six years, we have experimentally verified the expression of proteins predicted from genomic ortholog comparisons among 17 environmental and pathogenic bacteria. More exclusive relationships were observed among the expressed protein content of phenotypically related bacteria, which is indicative of the specific lifestyles associated with these organisms. Although genomic studies can establish relative orthologous relationships among a set of bacteria and propose a set of ancestral genes, our proteomics study establishes expressed lifestyle differences among conserved genes and proposes a set of expressed ancestral traits.

## Introduction

As a result of the numerous bacterial genome sequences currently available, the concept of a core genome–a set of orthologous genes commonly derived in bacterial genomic studies–is being used increasingly to explore genomic relationships among bacteria. For example, important insights into the origin of photosynthesis were recently obtained from the analysis of 892 core genes identified among 15 cyanobacteria genomes [1]. In another comparative genomic study of 4 magnetotactic bacteria, several unique genes from a core of 891 genes were identified as potentially important to the magnetic field sensing and taxis abilities of this group of prokaryotes [2]. A general observation from these studies is that the number of genes that make up the core genome depends on the number and diversity of organisms being compared [1]–[7].

While the use of the core genome concept has led to important insights into the evolution of bacterial species and identification of potentially important novel genes, there has been little discussion regarding actual expression of the core genome genes as proteins and the extent of this expression across the set of bacteria under study. The assumption that a gene will always produce a gene product, i.e., protein, is debatable as evidence suggests that genes are silenced by evolutionary mechanisms and as such, will not be expressed [8]. Thus, the expression of a core gene in one organism, but not in another can provide insight into the effects of both evolution and environmental pressures on the expressed phenotype. Yet the expression of identified genes within core genomes is rarely verified by experimental observation due to the extensive resources and rigorous experimental design required to do so.

We hypothesized that a core genome could be supported by a set of conserved proteins or core proteome, where the proteome is defined as the collection of structural and functional proteins actually present in the cell [9] and is thus a direct expression of cell phenotype [10], [11]. Herein, we show that examination of this hypothesis has important implications for a broad range of microbiological applications, such as determining the essentiality of genes derived from the core genome, deriving traits that correspond to a common ancestor (orthology) [4], [12], and on a more practical note, the direct identification of therapeutic and environmental targets or markers for additional characterization.

## Results

Enabled by a database of ∼967,000 experimentally determined unique peptides linked to specific protein information and publicly available genome sequences, we examined protein expression in a core genome of 17 bacteria. The peptide database is the result of high-throughput liquid chromatography mass spectrometry-based proteomics measurements obtained over six-years. Among the selected bacterial genotypes are the phyla Actinobacteria, Deinococcus-Thermus, Proteobacteria, and Cyanobacteria representing large evolutionary distances (based on 16S rDNA sequence alignment), as well as the species *Geobacter metallireducens* and *Geobacter sulfurreducens* that represent relatively short evolutionary distance. Notable bacteria include both pathogens, e.g., the *Yersinia* species and environmental bacteria, e.g., the metabolically diverse *Rhodobacter sphaeroides* and the ocean-dwelling *Peligibacter ubique*. We first identified a core genome by predicting orthologs among consecutively larger numbers of the bacteria (from 2 to 17), using the INPARANOID algorithm [13] in conjunction with BLAST [14]. Next, we searched our peptide databases for proteins that corresponded to the predicted orthologs. We required a minimum of two unique peptides identified using tandem mass spectrometry in conjunction with the SEQUEST algorithm [15] to confirm the presence of a protein in each organism.

### Identified Orthologs Supported by Protein Observation

**Figure 1:**
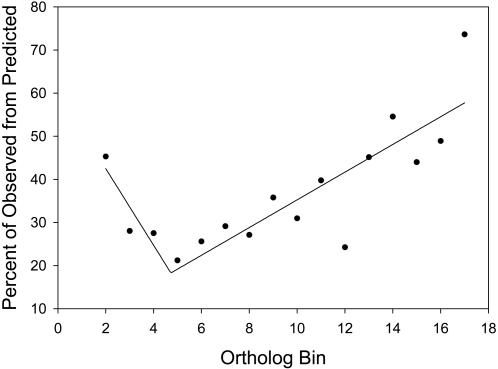
The relationship between the number of bacteria and the percent of observed proteins from predicted orthologs. The number of predicted orthologs represents the sum of orthologs identified *in-silico* from any combination of bacteria within the set. As the number of organisms increased from 5 to 17, proteomes among the individual bacteria converged to a set of conserved proteins, or core proteome.

**Figure 2:**
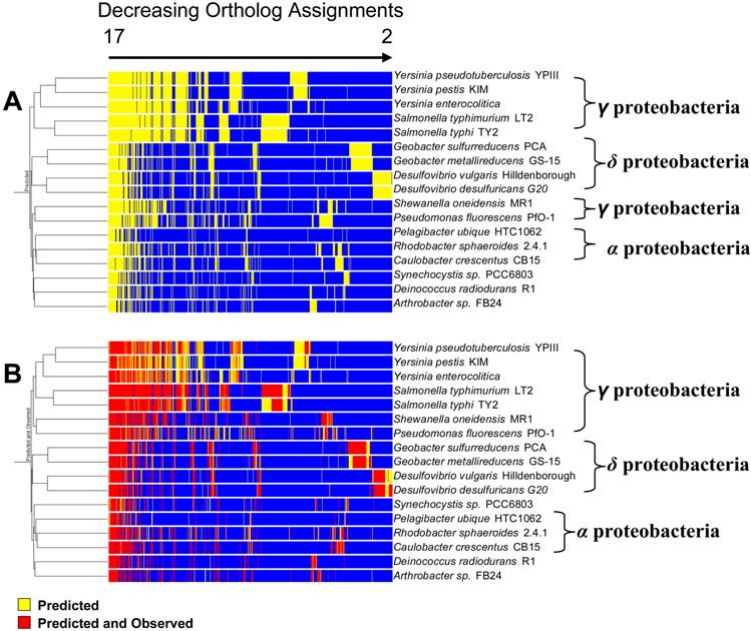
Predicted orthologs verified by proteomic observations. A) Orthologs were predicted between consecutively greater numbers of bacteria, beginning with all pairwise combinations and ending with all of the 17 bacteria. Clustered results reveal a core genome of 144 genes and more exclusive orthologs between bacteria of the same species. B) Observed protein orthologs measured using liquid chromatography tandem mass spectrometry were included and given greater weight than predicted orthologs only. Clustering resulted in improved agreement with phylogenetic predictions. 105 of the 144 core genes were verified by protein observations, which represent the core proteome for the set of bacteria.

On the basis of our experimental design, we surmised that the likelihood of observing a large percentage of proteins from our core genome would be small because of phylogenetic distance and the difference in environments required for growth. However, we were surprised that 105 (74%) of the 144 predicted orthologs that comprised our core genome had corresponding proteins expressed across all 17 bacteria (Fig 1). The percentage of observed proteins initially decreased as the number of selected bacteria increased from 2 to 5, but then increased as the number increased from 5 to 17 organisms. The former trend highlights the bias that results from selecting several pairs and a triplicate of bacteria that were related by the same genus, had similar growth environments, and had a proportionately large number of genomic orthologs identified among them (Fig. 2A). The latter trend suggests that the likelihood of proteins being observed and expressed in nature increases when they represent orthologs among multiple organisms (in this study, >5 bacteria).

As the number of organisms increased to 17, the proteomes of the individual organisms converged upon a set of conserved proteins; that is, the core proteome. Overall, our genomic comparison established the relative orthologous relationships among the 17 bacteria and proposed a set of possible ancestral genes assumed to be orthologous [16]. Comparative proteomic measurements were then used to establish expressed lifestyle differences among these relationships (Fig. 2B), as well as proposed a set of expressed traits associated with an ancestral bacteria.

**Figure 3:**
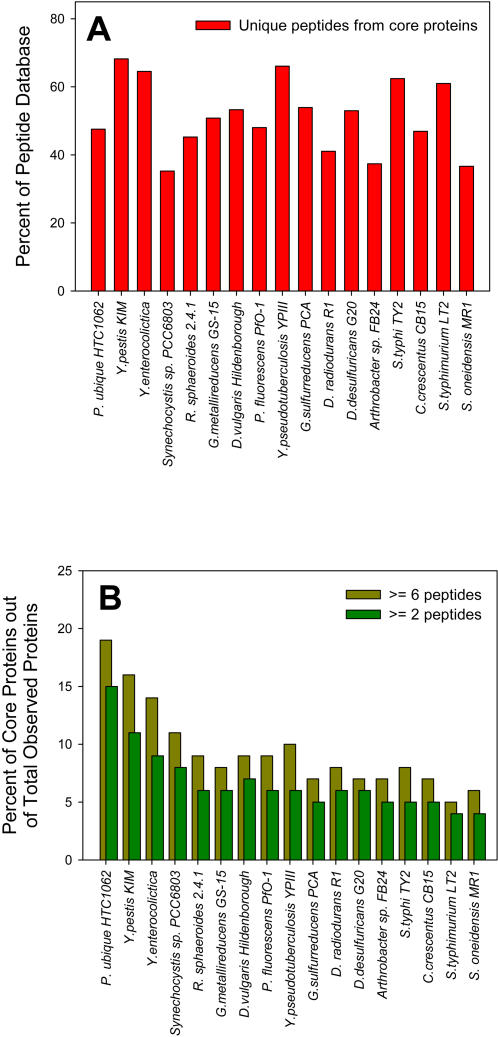
The analysis of peptides and their corresponding proteins identified within each bacterium's database of observed peptides. Organisms on the x-axis are sorted by proteome size (increasing). A) The percent of each proteome composed of peptides identifying core proteins predicted by the core genome. A significant percentage of each proteome was composed of these peptides, which suggests that they are regularly observed. B) The percentage of core proteins observed out of the total number of proteins identified by peptides within each proteome. As the number of peptides required to identify a protein increased (from 2 to 6 peptides), the percentage of core proteins out of the total number of observed proteins also increased.

Further evaluation of organism-specific proteomes revealed that a significant percentage of each proteome is composed of peptides representative of core proteome proteins (Fig. 3A). This observation was independent of the size of an organism's proteome. For example, ∼68% of the *Y. pestis* proteome, which had the second smallest set of observed peptides, and ∼62% of the *Salmonella enterica* subsp. *enterica* serotype Typhimurium LT2 *(S. typhimurium)* proteome, which had the second largest set of observed peptides, were composed of unique peptides from the core proteome. The set of peptides observed for each of the 17 organisms ranged in number from 11,870 to 103,873.

At the protein level, the percentage of observed proteins within each proteome that corresponded to core proteins increased as the number of peptides required to identify a protein was increased from 2 to 6 peptides (Fig. 3B). For example, 2547 proteins from the *R. sphaeroides* proteome database, including 141 proteins expressed from the core genome, were identified by 2 or more unique peptides (5.5%). Increasing the stringency from 2 to 6 peptides resulted in 1504 identified proteins composed of 129 core proteins (8.6%). For specific organisms such as *R. sphaeroides*, the observed proteome was constructed from as few as two culture conditions [17]; whereas, the observed proteome for *S. oneidensis* was generated from many (∼10) culture conditions. Based on the large percentage of observed proteins representative of the core proteome among a number of different culture conditions, we conclude that the core proteome is largely ubiquitous, in great abundance, and likely independent of culture condition.

### Functional Characterization of the Core Proteome

**Figure 4:**
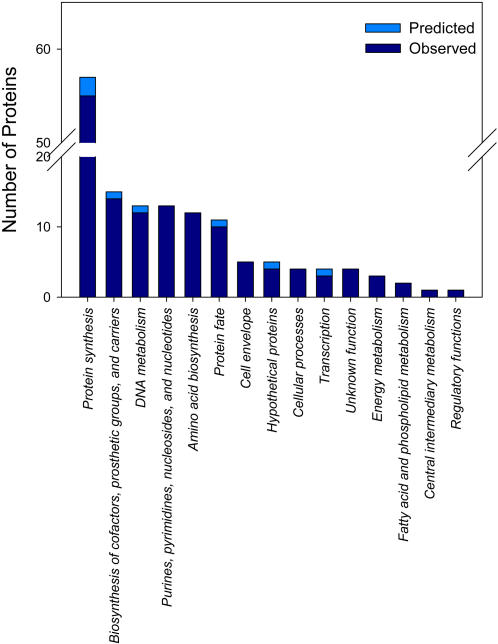
Functional categories (http://www.tigr.org/) assigned to the core genome and core proteome. The largest portion of the core proteome is involved in protein synthesis, which suggests the essentiality of these protein synthesis functions to freeliving bacteria. However, several proteins that were not well characterized according to functional category were also observed as part of the core proteome, which highlights the need for better characterization of these proteins.

In terms of functional assignments (www.tigr.org), a little over half (55%) of the proteins observed from the core genome are devoted to protein synthesis (Fig.4) and composed of ribosomal proteins and functional proteins associated with tRNA-aminoacylation, including methionyl-tRNA formyltransferase and methionyl-tRNA synthetase. Strikingly, ∼7% of the observed proteins have not been completely characterized with regard to functionality (Table S1). For example, a single protein in the core proteome was assigned a general regulatory function (Fig. 4). Designated as BipA /TypA, this protein belongs to the elongation factor GTPase superfamily and affects cellular function under multiple growth conditions [18]. Although BipA interacts with the ribosome and its GTPase activity is directly connected to the 70S ribosome charged with mRNA and aminoacylated tRNAs in *Escherichia coli*, its regulatory role remains unknown [18].

As another example, a recent review of previously identified core genes placed the *ybeB* gene near the top of a list that prioritized targets for experimentation [19]. Observed as a core protein within our bacterial set, this small protein (∼11 to ∼13 kDa) is a homolog of the Iojap plant protein. Mutations of this gene (e.g., in maize) lack expression of a plastid encoded RNA polymerase that exhibits some sequence similarity to bacterial RNA polymerases [20]. Recent evidence suggests this protein is associated with the 50S ribosomal subunit [21] and/or is involved in cell division [22]. Alignment of secondary structure predictions based on amino acid sequence [23], [24] for this protein indicate a high degree of symmetry around an alpha-helix structure (Fig. S1), similar to the secondary structure of the protein Calmodulin, which binds many protein targets and is involved in multiple cell functions. The presence of this protein and other such poorly characterized proteins across a broad spectrum of bacteria suggests a need for a greater understanding of basal functions associated with the free-living bacterial domain.

### Relative Comparison of Expressed Lifestyles

While certain basal functions across the set of 17 organisms are represented by conserved proteins within the core proteome, lifestyle differences become distinguishable outside the core (Fig. 2B). At the most exclusive regions where the search for ortholog assignments was between combinations of two organisms, large otholog clusters were identified for close phylogenetically related organisms. For example, a large cluster of 425 orthologs was identified as unique to the two *Geobacter* species in our bacterial set. Both organisms were cultured in the presence of Fe(III) citrate and 288 of the 425 identified orthologs had proteins expressed in both organisms, which demonstrates similar lifestyle responses to this electron acceptor. When *G. sulfurreducens* was cultured in the presence of fumarate and *G. metallireducens* in the presence of nitrate, differences in lifestyles associated with these two electron acceptors were also observed as a result of the different culture environments. Against the backdrop of unique orthologs, the lifestyle similarities and differences of these environmentally important metal reducing bacteria [25], [26] may serve as important environmental indicators for heavy metal reduction and as markers for monitoring the redox state required to maintain the immobilization of toxic metals.

**Figure 5:**
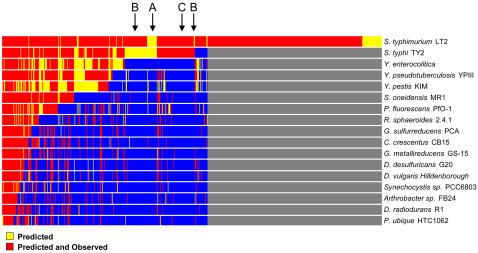
Predicted and observed orthologs shown relative to predicted and observed proteins in *S. typhimurium*. Approximately 50% of predicted proteins in *S. typhimurium* exhibited orthology (blue area) to at least one other bacterium in the set of bacteria. As expected, *S. typhi* had the greatest degree of predicted and observed orthology to *S. typhimurium*.. Certain regions of orthology are unique to the two serovars and include A) orthologs predicted only, B) orthologs predicted in both serovars, but observed in only one, and C) orthologs predicted and observed in both serovars. Categories A and B highlight proteins for future investigation as potential therapeutic targets.

Our comparative bacterial proteomic analysis lends itself to a unique reductionist approach for comparing lifestyles relative to a selected bacterium. Figure 5 shows individual proteomes of 16 of the organisms normalized relative to *S. typhimurium* (Fig. 5). In this comparison, the core proteome across all bacteria gives way to smaller subsets of common proteins among consecutively smaller numbers of bacteria. Forty proteins (Table S2), which included a unique RNase (mRNA degradation) and asparagine synthetase (multiple isozymes reported), were observed as common solely to the *Yersinia* species and the *Salmonella* serovars. With the addition of *S. oneidensis* MR1, the number of observed proteins common to the set dropped to 26 (Table S2). Among the 26 was the cell division protein ZipA, which is not highly conserved and present in only a limited number of gram-negative bacteria [27]. Ultimately, this type of comparison presents an opportunity for identifying proteins as unique environmental markers and potential broad-based or specific therapeutic targets.

In an initial demonstration, we applied this approach to the proteomes *S. typhimurium* and *Salmonella enterica* subsp. *enterica* serotype Typhi TY2 (*S. typhi*) to generate a set of potential therapeutic protein targets. The proteomes consisted of proteins extracted from several different cultures relevant to the pathogenicity of each organism, and a majority of the predicted proteins were observed for each organism. Although the *Salmonella* serovars exhibited 84% genome hybridization similarity [28], differences in their relative proteomic content were revealed by (Fig. 5): 1) unique orthologs predicted for both organisms, but not observed; 2) predicted orthologs uniquely observed, but in only one of the two serovars; and 3) predicted orthologs uniquely observed in both organisms. Proteins in the first category are of less interest as there are no experimentally observed gene products, i.e., proteins. Proteins in the second category represent an important group of potential therapeutic targets because proteome measurements delineated one organism from the other even though genomic comparisons predicted ortholog similarity. For example, 11 proteins were observed in *S. typhi*, but not in *S. typhimurium* and have putative annotations with predicted localizations in the inner membranes and cytoplasm. One of these 11 proteins is designated as a chaperone for the stabilization of fimbriae, important virulent proteins involved in the attachment of a pathogen to a host cell [29]. While the predicted orthologs from genomic comparison separate the *Salmonella* species from the rest of the bacteria in the third category, observation of gene products for each of these orthologs makes them particularly attractive as potential therapeutic targets for both serovars. A number of putative cytoplasmic, inner membrane and periplasmic proteins, as well as proteins from several characterized operons (e.g., *ssa*, *sse*, and *inv*) that contain known virulence genes make up this third category. We expect that future addition of organisms to our proteomic comparison will narrow this list to a subset of potential targets that have an even greater potential of therapeutic value.

## Discussion

Our peptide-centric proteomic measurements experimentally demonstrate the existence of a core set of genes that define bacterial life for a diverse set of bacteria. We suspect that the number of protein encoding genes within the core genome is dependent on the number of bacteria compared, but the expression of these genes as proteins is relatively inflexible to culture condition. As such, the core proteome represents an important set of expressed conserved proteins that have survived repeated speciation events.

An important implication of the core proteome is its essentiality to the set of bacteria studied and to the bacterial domain as a whole. In comparative genomic studies, gene essentiality is a common theme [6], [30] and is often discussed in the context of environment [31] where genes in a single species are essential for one environment, but nonessential for another. This essentiality is especially pertinent to free-living microbes, where a species must be able to subsist within a range of environmental fluctuations. For host-dependent microbes a relatively stable environment reduces the genome size compared to free-living bacteria; thereby, reducing the need for a large array of biological functions [32]. Numerous characterization studies of these minimal genomes in terms of essentiality have been performed [3], [6], [30], [31], [33], [34].

In evaluating gene essentiality in the broader context, we conclude that essentiality of a gene for bacterial life depends in part on gene conservation among organisms [35], as well as on the expression of these genes as proteins regardless of environment. In generating our core genome, we emphasized gene essentiality by including *P. ubique*, which has the fewest number of predicted protein encoding genes relative to its genome size of any free-living organism [36] and by requiring gene conservation across a phylogenetically diverse bacterial set. Our observed core proteome also suggests the need to perform random mutagenesis on individual species within our selected set of bacteria to further evaluate gene essentiality [33], [37] in the broader context. Nevertheless, essentiality of many of the translated genes within our core proteome has been empirically shown in other model organisms, such as *Escherichia coli* K12 MG1655 (Table S3) [38] and *S. typhimurium* [39] (Table S3).

In this study, observation of an ortholog in one organism and not another is likely a result of phenotypic plasticity, i.e., the ability of an organism to change its phenotype based on environment [40]. (Admittedly, the lack of protein observations in some organisms, for example *Y. pestis*, could also result from the more modest set of experimental results as compared to the other organisms.) The effect of plasticity on the hierarchical clustering of orthologs is illustrated in Figure 2. Figure 2A predicts a common set of genes at each internal node (possibly ancestral genes), and the order of clustered organisms is in reasonable agreement with established phylogeny. Conversely, the clustering of expressed proteins from predicted orthologs (Fig. 2B) represents the union of phenotypic traits (possibly common ancestral traits) at internal nodes, with the core proteome representing the root node.

As one progresses from the internal nodes toward the root node within the hierarchical structure presented in Figure 2B, one might speculate that the reduced degree of phenotypic plasticity suggests these common phenotypic traits are increasingly independent of the current niches of our bacterial set and rather are representative of a primordial niche. Ultimately, understanding phenotypic plasticity will be important to researchers interested in designing synthetic organisms for the purpose of biofuel production, pollution clean-up etc. [41], as a baseline of phenotypic traits will be required. To accomplish these designs, a set of relatively non-plastic phenotypic traits needs to be identified, which cannot be determined by genomics alone.

## Materials and Methods

### Organisms and Culture Conditions

The bacteria used for this study were previously cultured by several laboratories interested in the proteomic characterization of a given organism. Samples were kindly generated (Acknowledgements) for the purpose of developing an observed reference peptide database for each organism, utilizing the high-throughput proteomic capabilities present at Pacific Northwest National Laboratory, Richland WA. Many of the laboratory culture conditions have previously been published in connection with the primary proteomics work being conducted at Pacific Northwest National Laboratory [17], [42]–[55].

### Sample Preparation

Either an established [52] or optimized [17], [42]–[56] protein extraction protocol was applied to each cell culture. In brief, global (total), insoluble, and soluble protein digests were extracted from lysed cultures that were washed and suspended in 100 mM NH_4_HCO_3_, pH 8.4 buffer. For global extracts, proteins were denatured and reduced by adding urea, thiourea, and dithiothreitol (DTT) followed by incubation at ∼60°C for ∼30 min. Following incubation, the global protein samples were diluted to reduce salt concentration then proteolytic digested, at 37°C for ∼4 h, using sequencing grade trypsin (Roche, Indianapolis, IN) at a ratio of 1 unit per 50 units of protein (1 unit = ∼1 µg of protein). Following incubation, digested samples were desalted using an appropriately sized C-18 SPE column (Supelco, St. Louis, MO) and a vacuum manifold. The collected peptides were concentrated to a final volume ranging from 50 µl to 100 µl and measured using the BCA assay (Pierce Chemical Co., Rockfort, IL) according to the manufacturer's instructions.

For the insoluble protein digest, the cell lysate was ultracentrifuged at 4°C and 100,000 rpm for 10 min. The resulting supernatant that contained soluble proteins was separated from the pellet and retained for digestion as previously described for the global extraction. The pellet was washed by suspending it in 100 mM NH_4_HCO_3_, pH 7.8, using mild sonication and then ultracentrifuged at 100,000 rpm for 5 min, again at 4°C. Following centrifugation, the pellet was resuspended in a solubilizing solution that contained urea, thiourea, 1% CHAPS in 50 mM NH_4_HCO_3_, pH 7.8. An aliquot of 50 mM DTT solution was also added to final concentration of 5 mM. The insoluble protein sample was then incubated and digested as described above with the exception that a 50 mM NH_4_HCO_3_, pH 7.8 buffer was used for the dilution step. Following proteolytic digestion, the pH of the sample was slowly lowered to <4.0 by adding small volumes (1 µl to 2 µl) of 20% formic acid. Removal of salts and detergent was performed using either an appropriately sized strong cation exchange (SCX) or solid phase extraction column (Supelco, St. Louis, MO) and vacuum manifold. Peptides were then concentrated and their concentration measured as described above.

### Database Generation and Filtering

Databases of observed peptides were generated according to an established protocol [52], [57], [58]. In brief, peptides from the global, insoluble, and soluble digests were fractionated (25 to 100 fractions each) using high resolution reversed-phase SCX high pressure liquid chromatography (HPLC). The HPLC system was operated in an exponential gradient mode with mobile phase B (0.1% TFA in 90% ACN and 10% water) replacing mobile phase A (0.2% acetic acid, 0.05% TFA in water) 10 min after sample injection, which was accomplished by using an in-house mixer, capillary column selector, and sample loop.

From each collected fraction, a consistent mass of peptides were analyzed by reversed phase HPLC coupled on line to an ion trap mass spectrometer (LCQ and/or LTQ ThermoFischer, San Jose, CA) operated in a data-dependent MS/MS mode. MS/MS spectra were analyzed using the SEQUEST algorithm [15] in conjunction with publicly available predicted protein sequences from the appropriate genome sequence. Preliminary filtering of identified peptides was performed using a minimum cross-correlation cut-off (*Xcorr*) of either 1.9, 2.2, or 3.75 for 1+, 2+, or 3+ charge states, respectively, for fully tryptic (peptides that contained either an arginine or lysine at the site of cleavage), partially tryptic, and non-tryptic peptides. All peptides were a minimum of 6 amino acids long. For this specific study, peptides in the databases were further filtered using a PeptideProphet [59] score of at least 0.90. Note that PeptideProphet calculates the probability that a peptide sequence has been correctly assigned [59]. Although database dependent, filtering on a PeptideProphet score of 0.95 roughly corresponds to a ∼5% false discovery rate based on reverse database searching techniques [47], [60].

### Ortholog Identification

Orthologs were idendified using INPARANOID v.1.35 [13]. This program uses BLAST [14] to compare the complete set of protein sequences from one genome with that of another, and identifies the reciprocal best hits. We set the parameters to utilize the BLOSUM62 matrix and a minimum bit score of 30, and we required that the BLAST alignment cover at least 50% of both proteins. The resulting ortholog tables were analyzed by Perl scripts to identify complete ortholog graphs (http://mathworld.wolfram.com/CompleteGraph.html) where the nodes of the graphs are the proteins and the edges are the INPARANOID ortholog connections. Complete ortholog graphs have *n* nodes and 
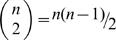
 edges, where *n* is the number of input genomes.

## Supporting Information

Figure S1 Aligned secondary structure predictions based on amino acid sequence for YbeB. A conserved and symmetrical secondary structure was predicted for this protein indicative of a possible binding protein. H-helix; E-extended strand
(0.23 MB PDF)

Table S1 Core proteins described as having a general functional characterization or no functional characterization.
(0.14 MB PDF)

Table S2 Conserved proteins from different sub-sets of bacteria relative to *S. typhimurium*.
(0.12 MB PDF)

Table S3 Core proteome proteins observed in *E. coli* K12 MG1655 and *S. typhimurium* and their published genes noted as essential. (Source: Gerdes, et al. 2003. *J. Bacteriol.* 185(19):5673–5684; Knuth, et al. 2004. *Mol Microbiol.* 51(6):1729–1744)
(0.25 MB PDF)
