# Supplementary material for: Comparative Bacterial Proteomics: Analysis of the Core Genome Concept
Source: PLoS One. 2008 Feb 6;3(2):e1542. doi: 10.1371/journal.pone.0001542 (PMC2213561; doi:10.1371/journal.pone.0001542)
Supplement: Figure S1 — Aligned secondary structure predictions based on amino acid sequence for YbeB. A conserved and symmetrical secondary structure was predicted for this protein indicative of a possible binding protein. H-helix; E-extended strand (0.23 MB PDF) [file pone.0001542.s001.pdf]

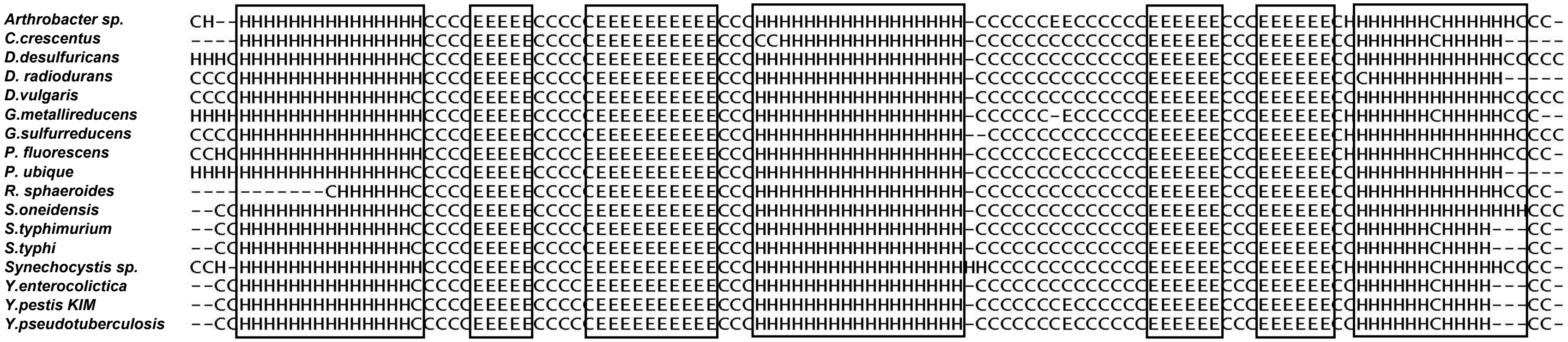

Arthrobacter sp. CH--HHHHHHHHHHHHHHHHCCCCEEEEECCCCCEEEEEEEEEEECCCHHHHHHHHHHHHHHHHHH-CCCCCCEECCCCCCEEEEEECCCEEEEEECHHHHHHHCHHHHHHCCC-
C.crescentus ----HHHHHHHHHHHHHHHHCCCCEEEEECCCCCEEEEEEEEEEECCCCCHHHHHHHHHHHHHHHH-CCCCCCCCCCCCCCEEEEEECCCEEEEEECCHHHHHHCHHHHH-----
D.desulfuricans HHHCHHHHHHHHHHHHHHHCCCCCEEEEECCCCCEEEEEEEEEEECCCHHHHHHHHHHHHHHHHHH-CCCCCCCCCCCCCCEEEEEECCCEEEEEECCHHHHHHHHHHHHCCCCC
D. radiodurans CCCCHHHHHHHHHHHHHHHHCCCCEEEEECCCCCEEEEEEEEEEECCCHHHHHHHHHHHHHHHHHH-CCCCCCCCCCCCCCEEEEEECCCEEEEEECCCHHHHHHHHHHH-----
D.vulgaris CCCCHHHHHHHHHHHHHHHCCCCCEEEEECCCCCEEEEEEEEEEECCCHHHHHHHHHHHHHHHHHH-CCCCCCCCCCCCCCEEEEEECCCEEEEEECCHHHHHHHHHHHHCCCCC
G.metallireducens HHHHHHHHHHHHHHHHHHHHCCCCEEEEECCCCCEEEEEEEEEEECCCHHHHHHHHHHHHHHHHHH-CCCCCC-ECCCCCCEEEEEECCCEEEEEECHHHHHHHCHHHHHCCC--
G.sulfurreducens CCCCHHHHHHHHHHHHHHHCCCCCEEEEECCCCCEEEEEEEEEEECCCHHHHHHHHHHHHHHHHHH--CCCCCCCCCCCCCEEEEEECCCEEEEEECHHHHHHHHHHHHHHCCCC
P. fluorescens CCHCHHHHHHHHHHHHHHHHCCCCEEEEECCCCEEEEEEEEEEEECCCHHHHHHHHHHHHHHHHHH-CCCCCCCECCCCCCEEEEEECCCEEEEEECHHHHHHHCHHHHHCCCC-
P. ubique HHHHHHHHHHHHHHHHHHHCCCCCEEEEECCCCCEEEEEEEEEEECCCHHHHHHHHHHHHHHHHHH-CCCCCCCCCCCCCCEEEEEECCCEEEEEECCHHHHHHHHHHHH-----
R. sphaeroides -------------CHHHHHHCCCCCEEEEECCCCCEEEEEEEEEEECCCHHHHHHHHHHHHHHHHHH-CCCCCCCCCCCCCCEEEEEECCCEEEEEECCHHHHHHHHHHHHCCCC-
S.oneidensis --CCHHHHHHHHHHHHHHHCCCCCEEEEECCCCEEEEEEEEEEEECCCHHHHHHHHHHHHHHHHHH-CCCCCCCCCCCCCCEEEEEECCCEEEEEECCHHHHHHHHHHHHHHCCC
S.typhimurium --CCHHHHHHHHHHHHHHHCCCCCEEEEECCCCCEEEEEEEEEEECCCHHHHHHHHHHHHHHHHHH-CCCCCCCCCCCCCCEEEEEECCCEEEEEECCHHHHHHCHHHH---CC-
S.typhi --CCHHHHHHHHHHHHHHHCCCCCEEEEECCCCCEEEEEEEEEEECCCHHHHHHHHHHHHHHHHHH-CCCCCCCCCCCCCCEEEEEECCCEEEEEECCHHHHHHCHHHH---CC-
Synechocystis sp. CCH-HHHHHHHHHHHHHHHHCCCCEEEEECCCCEEEEEEEEEEEECCCHHHHHHHHHHHHHHHHHHHCCCCCCCCCCCCCEEEEEECCCEEEEEECHHHHHHHCHHHHHCCCC-
Y.enterocolictica --CCHHHHHHHHHHHHHHHCCCCCEEEEECCCCEEEEEEEEEEEECCCHHHHHHHHHHHHHHHHHH-CCCCCCCCCCCCCCEEEEEECCCEEEEEECCHHHHHHCHHHH---CC-
Y.pestis KIM --CCHHHHHHHHHHHHHHHCCCCCEEEEECCCCCEEEEEEEEEEECCCHHHHHHHHHHHHHHHHHH-CCCCCCCECCCCCCEEEEEECCCEEEEEECCHHHHHHCHHHH---CC-
Y.pseudotuberculosis --CCHHHHHHHHHHHHHHHCCCCCEEEEECCCCCEEEEEEEEEEECCCHHHHHHHHHHHHHHHHHH-CCCCCCCECCCCCCEEEEEECCCEEEEEECCHHHHHHCHHHH---CC-
