# Supplementary material for: Comparative Bacterial Proteomics: Analysis of the Core Genome Concept
Source: PLoS One. 2008 Feb 6;3(2):e1542. doi: 10.1371/journal.pone.0001542 (PMC2213561; doi:10.1371/journal.pone.0001542)
Supplement: Table S1 — Core proteins described as having a general functional characterization or no functional characterization. (0.14 MB PDF) [file pone.0001542.s002.pdf]

Table S1. Core proteins described as having a general functional characterization or no functional characterization.

Protein	Organism	Approximate DNA Coordinates	Locus Tag*	Gene Associated Description
prot31 (Unknown Function)	*Arthrobacter sp.* FB24	2676632…2678221 (-)	Arth_2389	GTP1/OBG subdomain PFAM
	C. crescentus CB15	331907..332971 (-)	CC0315	GTP-binding protein CgtA
	D. radiodurans R1	86474…87790 (-)	DR0084	GTP-binding protein Obg
	D. desulfuricans G20	2696935…2698218 (-)	Dde_2690	GTP-binding protein, GTP1/OBG family
	D. vulgaris Hilldenborough	1018596…1019696 (+)	DVU0929	GTP-binding protein, GTP1/OBG family
	G. metallireducens GS-15	3597639…3598655 (+)	Gmet_3197	GTP-binding protein, GTP1/OBG family
	G. sulfurreducens PCA	3519000…3520016 (-)	GSU3213	GTP-binding protein, GTP1/OBG family
	P. fluorescens PfO-1	5477922…5479145 (-)	Pfl_4858	GTP-binding protein, GTP1/Obg family
	P. ubique HTC1062	221539…222522 (+)	SAR11_0222	GTP-binding protein
	R. sphaeroides 2.4.1	17904…18932 (+)	RSP3822	ATP/GTP-binding site motif A (P-loop):GTP1/OBG family
	S. oneidensis MR-1	3805701…3806867 (-)	SO3649	GTP-binding protein, GTP1/Obg family
	S. typhi TY2	3310236…3311408 (-)	t3218	Probable GTP-binding protein
	S. typhimurium LT2	3470219…3471391	STM3301	Putative GTP-binding protein
	Synechocystis sp. PCC6803	397037…398143 (+)	slr1090	GTP-binding protein
	Y. enterocolictica	489388…490560 (+)	YE0421	Putative GTP-binding factor
	Y. pestis KIM	758194…759366 (+)	y0675	Putative GTP-binding factor
	Y. pseudotuberculosis YPIII	551563…552735 (+)	YPTB0467	Putative GTP-binding protein
prot36 (Unknown Function)	*Arthrobacter sp.* FB24	2671314…2671715 (-)	Arth_2383	Iojap-related protein TIGR
	C. crescentus CB15	3676177…3676518 (+)	CC3432	Iojap-related protein
	D. radiodurans R1	2588246…2588596 (-)	DR2580	conserved hypothetical protein
	D. desulfuricans G20	1813661…1814056 (+)	Dde_1756	Iojap-related protein
	D. vulgaris Hilldenborough	1702850…1703254 (+)	DVU1618	Iojap-related protein
	G. metallireducens GS-15	3601811…3602206 (+)	Gmet_3201	Iojap-related protein
	G. sulfurreducens PCA	3515275…3515670 (-)	GSU3209	Iojap-related protein
	P. fluorescens PfO-1	5600475…5600969 (-)	Pfl_4972	Hypothetical protein
	P. ubique HTC1062	225411…225761 (+)	SAR11_0226	Iojap-related protein
	R. sphaeroides 2.4.1	2616422…2616742 (+)	RSP0865	Iojap protein family
	S. oneidensis MR-1	1213093…1213422 (-)	SO1170	Iojap domain protein
	S. typhi TY2	2291579…2291896 (+)	t2225	Conserved hypothetical protein (ybeB)
	S. typhimurium LT2	704426…704743 (-)	STM0642	Homolog of plant Iojap protein (ybeB)
	Synechocystis sp. PCC6803	1245395…1245859 (+)	slr1886	Hypothetical protein
	*Y. enterocolictica***	3271557…3271874 (+)	YE3000	Hypothetical protein

	Y. pestis KIM**	1324183…1324500 (-)	y1180	Hypothetical protein
	Y. pseudotuberculosis YPIII	1316981…1317298 (-)	YPTB1099	Conserved hypothetical protein
**prot325 (Hypothetical Protein)**	*Arthrobacter sp.* FB24	2513295…2514071 (-)	Arth_2234	Conserved hypothetical protein
	C. crescentus CB15	3658975…3659691 (-)	CC3415	Conserved hypothetical protein
	D. radiodurans R1	1535576…1536304 (-)	DR1520	Conserved hypothetical protein
	D. desulfuricans G20	2040355…2041071 (+)	Dde_1997	Conserved hypothetical protein
	D. vulgaris Hilldenborough	1764117..1764866 (+)	DVU1685	Conserved hypothetical protein
	G. metallireducens GS-15	3800542…3801733 (+)	Gmet_3387	Conserved hypothetical protein
	G. sulfurreducens PCA	477066…477848 (-)	GSU0446	Conserved hypothetical protein
	P. fluorescens PfO-1	5951311…5952030 (+)	Pfl_5288	Hypothetical protein
	P. ubique HTC1062**	143525…144226 (-)	SAR11_0136	Conserved hypothetical protein
	R. sphaeroides 2.4.1	2765413…2766186 (-)	RSP1007	Conserved hypothetical protein
	S. oneidensis MR-1	850577…851308 (-)	S0832	Conserved hypothetical proteins
	S. typhi TY2	3097369…3098100 (+)	t3006	Conserved hypothetical protein
	S. typhimurium LT2	3256140…3256919 (+)	STM3094	Putative cytoplasmic protein (yggJ)
	Synechocystis sp. PCC6803	108290…109201 (+)	slr0722	Hypothetical protein
	Y. enterocolictica	3740817…3741548 (+)	YE426	Hypothetical protein
	Y. pestis KIM**	3654517…3655248 (+)	y3320	Hypothetical protein
	Y. pseudotuberculosis YPIII	3772297…3773028 (+)	YPTB3206	Conserved hypothetical protein
**prot330 (Uknown Function)**	*Arthrobacter sp.* FB24	2519298…2521151 (-)	Arth_2240	Small GTP-binding protein domain (lepA)
	C. crescentus CB15	1165328..1167274 (-)	CC1034	GTP-binding protein LepA
	D. radiodurans R1	1153825…1155645 (-)	DR1145	GTP-binding elongation factor family protein LepA
	D. desulfuricans G20	2802606…2804411 (-)	Dde_2817	Small GTP-binding protein domain:GTP-binding protein
	D. vulgaris Hilldenborough	782190…783995 (+)	DVU0703	GTP-binding protein LepA
	G. metallireducens GS-15	1980477…1982366 (+)	Gmet_1766	GTP-binding protein LepA
	G. sulfurreducens PCA	1377368…1379170 (+)	GSU1266	GTP-binding protein LepA
	P. fluorescens PfO-1	1153445…1155241 (+)	Pfl_0992	GTP-binding protein lepA
	P. ubique HTC1062	438405…440213 (+)	SAR11_0444	GTP-Binding protein lepA
	R. sphaeroides 2.4.1	681644…683443 (-)	RSP2088	GTP-binding elongation factor
	S. oneidensis MR-1	1402041…1403831 (+)	SO1346	GTP-binding protein (LepA)
	S. typhi TY2	312191…313990 (+)	t0274	GTP-binding protein LepA
	S. typhimurium LT2	2726772…2728571 (-)	STM2583	GTP-binding elongation factor (lepA)
	Synechocystis sp. PCC6803	3564838…3566649 (+)	slr0604	GTP-binding protein
	Y. enterocolictica	1134410…1136209 (+)	YE1015	GTP-binding elongation factor (lepA)
	Y. pestis KIM	1438342…1440141 (+)	y1295	GTP-binding elongation factor (lepA)

	Y. pseudotuberculosis YPIII	3422285…3424084 (-)	YPTB1099	Putative GTP-binding elongation factor (lepA)
**prot371 (Hypothetical Protein)**	*Arthrobacter sp.* FB24	2593536…2594291 (-)	Arth_2304	Protein of unknown function DUF28
	C. crescentus CB15	3504459…3505214 (-)	CC3243	Conserved hypothetical protein
	D. radiodurans R1	2550845…2551579 (-)	DR2548	Conserved hypothetical protein
	D. desulfuricans G20	2342035…2342781 (-)	Dde_2325	Protein of unknown function DUF28
	D. vulgaris Hilldenborough	2352787…2353530 (-)	DVU2259	Conserved hypothetical protein
	G. metallireducens GS-15	817626…818369 (+)	Gmet_0743	Hypothetical protein
	G. sulfurreducens PCA	1163086…1163829 (+)	GSU1074	Conserved hypothetical protein
	P. fluorescens PfO-1	4969166...4969912 (-)	Pfl_4410	Hypothetical UPF0082 protein
	P. ubique HTC1062	578863…579588 (+)	SAR11_0592	Domain of unknown function DUF28
	R. sphaeroides 2.4.1	2393628…2394374 (-)	RSP0655	Conserved hypothetical
	S. oneidensis MR-1	2540604…2541350 (-)	SO2432	Expressed protein of unknown function DUF28
	S. typhi TY2	1066996…1067736 (+)	t0978	Conserved hypothetical protein (yebC)
	S. typhimurium LT2	1993786…1994526 (-)	STM1899	Putative cytoplasmic protein (yebC)
	Synechocystis sp. PCC6803**	22572036…2257964 (+)	slr0989	Hypothetical protein
	Y. enterocolictica	2583739…2584482 (-)	YE2395	Hypothetical protein
	Y. pestis KIM	2484824…2485579 (-)	y2255	Hypothetical protein
	Y. pseudotuberculosis YPIII	2402812…2403555	YPTB2038	Conserved hypothetical protein
**prot738 (Hypothetical Protein)**	*Arthrobacter sp.* FB24	2809834..2810412 (+)	Arth_2509	Putative methyltransferase
	C. crescentus CB15	243490…244056 (-)	CC0227	Conserved hypothetical protein
	D. radiodurans R1	655597…656157 (-)	DR0643	N-6 adenine-specific DNA restriction methylase, Putative
	D. desulfuricans G20	1834501…1835070 (+)	Dde_1782	Conserved hypothetical protein
	D. vulgaris Hilldenborough	1599751…1600323 (+)	DVU1531	Methyltransferase, Putative
	G. metallireducens GS-15	1974741…1975307 (-)	Gmet_1760	Hypothetical protein
	G. sulfurreducens PCA	1345954…1346517 (-)	GSU1244	Methyltransferase, Putative
	P. fluorescens PfO-1	6000676…6001284 (+)	Pfl_5340	Methyltransferase, Putative
	P. ubique HTC1062	157024…157590 (+)	SAR11_0154	N6-adenine-specific methylase
	R. sphaeroides 2.4.1	2651080…2651634 (+)	RSP0901	N-6 Adenine-specific DNA methylase
	S. oneidensis MR-1	4782794…4783441 (+)	SO4587	Putative methylase involved in ubiquinone biosynthesis
	S. typhi TY2	4089701…4090297 (-)	t3950	Conserved hypothetical protein
	S. typhimurium LT2	3740347…3740943 (+)	STM3572	Putative methyltransferase
	Synechocystis sp. PCC6803**	2385351…2385911 (+)	slr0383	Hypothetical protein
	*Y. enterocolictica***	259672…260226 (-)	YE0224	Hypothetical protein
	Y. pestis KIM**	463268…463930 (-)	y0415	Hypothetical protein

	Y. pseudotuberculosis YPIII	262318…262980 (-)	YPTB0220	Conserved hypothetical protein
**prot774 (Unknown Function)**	*Arthrobacter sp.* FB24	2896748..2897452 (-)	Arth_2580	Ham1-like protein TIGR
	C. crescentus CB15	156966…157550 (-)	CC0146	Ham1 family protein
	D. radiodurans R1	179684…180286 (+)	DR0179	Conserved hypothetical protein
	D. desulfuricans G20	511225…511854 (-)	Dde_0504	Ham1-like protein
	D. vulgaris Hilldenborough	3307131…3307754 (+)	DVU3154	HAM1 family protein
	G. metallireducens GS-15	2090602…2091192 (-)	Gmet_1875	HAM1-like protein
	G. sulfurreducens PCA	1960195…1960794 (-)	GSU1794	HAM1 protein
	P. fluorescens PfO-1	5487966…5988562 (+)	Pfl_5326	HAM1 protein homolog
	P. ubique HTC1062	361572…362174 (+)	SAR11_0370	HAM1 protein homolog
	R. sphaeroides 2.4.1	2994982…2995593 (+)	RSP1222	Putative Ham1p_like, Ham1 family
	S. oneidensis MR-1	3500363…3500980 (+)	SO3358	Non-canonical purine NTP pyrophosphatase, rdgB/HAM1
	S. typhi TY2	3103707…3104300 (+)	t3015	Conserved hypothetical protein
	S. typhimurium LT2	3262528…3263121 (+)	STM3103	Putative Xanthosine triphosphate pyrophosphatase
	Synechocystis sp. PCC6803	2151606…2152190 (+)	slr0402	Hypothetical protein
	Y. enterocolictica	3750245…3750838 (+)	YE3437	Putative ribosomal protein
	Y. pestis KIM	3663573…3664244 (+)	y3331	Putative ribosomal protein
	Y. pseudotuberculosis YPIII	3781431…3782024 (+)	YPTB3217	Possible Xanthosine triphosphate pyrophosphatase
**prot901 (Regulatory Function)**	*Arthrobacter sp.* FB24	3165359..3167287 (-)	Arth_2817	Small GTP-binding protein domain:GTP-binding protein (TypA_BipA)
	C. crescentus CB15	811916…813748 (-)	CC0741	elongation factor Tu family protein
	D. radiodurans R1	1206001…1207782 (-)	DR1198	GTP-binding elongation factor family protein TypA/BipA
	D. desulfuricans G20	1551395…1553239 (-)	Dde_1533	Small GTP-binding protein domain:GTP-binding protein TypA
	D. vulgaris Hilldenborough	2323364…2325205 (+)	DVU2231	GTP-binding protein TypA
	G. metallireducens GS-15	3397598…3399394 (+)	Gmet_3013	GTP-binding protein
	G. sulfurreducens PCA	533056…534852 (+)	GSU0500	GTP-binding protein TypA
	P. fluorescens PfO-1	391491…393311 (+)	Pfl_0347	GTP-binding protein TypA/BipA
	P. ubique HTC1062	168376…170202 (+)	SAR11_0165	GTP-binding protein, TypA
	R. sphaeroides 2.4.1	2178615…2180435 (+)	RSP0448	EF-Tu; elongation factor Tu
	S. oneidensis MR-1	4600874…4602685 (-)	SO4408	virulence regulator BipA
	S. typhi TY2	3711782…3713605 (-)	t3613	GTP-binding elongation factor family protein (typA)
	S. typhimurium LT2	4217384…4219207 (+)	STM4009	GTP-binding protein (typA)
	Synechocystis sp. PCC6803	204604…206397 (+)	slr1105	GTP-binding protein TypA/BipA
	Y. enterocolictica	38529…40352 (+)	YE0029	Putative GTP-binding factor
	Y. pestis KIM	4221887…4223710 (-)	y3803	Putative GTP-binding factor

	Y. pseudotuberculosis YPIII	34478…36301 (+)	YPTB0025	Putative GTPase (bipA)
**prot902 (Hypothetical Protein)**	*Arthrobacter sp.* FB24	3185803..3186909 (-)	Arth_2831	GTP-biniding protein YchF
	C. crescentus CB15	499915…501015 (-)	CC0479	GTP-binding protein, YchF family
	D. radiodurans R1	1391767…1392864 (+)	DR1386	conserved hypothetical protein
	D. desulfuricans G20	1752704…1753804 (-)	Dde_1698	Conserved hypothetical protein
	D. vulgaris Hilldenborough	1503735…1504835 (-)	DVU1429	GTP-binding protein
	G. metallireducens GS-15	3223418…3224512 (-)	Gmet_2845	GTP-binding protein
	G. sulfurreducens PCA	700226…701320 (+)	GSU0664	GTP binding protein YchF
	P. fluorescens PfO-1	5359504…5360604 (+)	Pfl_4756	GTP-binding protein YchF
	P. ubique HTC1062	109411…110484 (+)	SAR11_0097	Predicted GTPase, Probable translation factor
	R. sphaeroides 2.4.1	2577892…2578989 (+)	RSP0832	Putative GTP-binding protein
	S. oneidensis MR-1	1228241…1229332 (+)	SO1185	GTPase/translation factor
	S. typhi TY2	1176621…1177712 (-)	t1092	Putative ATP/GTP-binding protein
	S. typhimurium LT2	1881818…1882909 (+)	STM1784	Putative GTP-binding protein
	Synechocystis sp. PCC6803	1520009…1521100 (-)	sll0245	Probable GTP binding protein
	Y. enterocolictica	2629139…2630230 (+)	YE2439	Putative GTP-binding protein
	Y. pestis KIM	2533619…2534710 (+)	y2298	Putative GTP-binding protein
	Y. pseudotuberculosis YPIII	2354015…2355106 (-)	YPTB3217	Conserved hypothetical protein
**prot1177 (Hypothetical Protein)**	*Arthrobacter sp.* FB24	1311164..1312177 (+)	Arth_1206	Protein of unknown function UPF0011 TIGR
	C. crescentus CB15	154912…155784 (-)	CC0144	Tetrapyrrole methylase family protein
	D. radiodurans R1	650694…651653 (-)	DR0636	Conserved hypothetical protein
	D. desulfuricans G20	1122376…1123209 (-)	Dde_1092	Protein of unknown function UPF0011
	D. vulgaris Hilldenborough	922377…923204 (-)	DVU0832	Tetrapyrrole methylase family protein
	G. metallireducens GS-15	3246398…3247246 (-)	Gmet_2861	Hypothetical protein
	G. sulfurreducens PCA	683565…684425 (+)	GSU0653	Tetrapyrrole methylase family protein
	P. fluorescens PfO-1	1348994…1347876 (-)	Pfl_4683	Decarboxylase family protein
	P. ubique HTC1062	363342…364205 (+)	SAR11_0372	Possible methylase or methyltransferase
	R. sphaeroides 2.4.1	401856…402743 (+)	RSP1813	Conserved hypothetical protein
	S. oneidensis MR-1	306578…307423 (+)	SO0301	Methyltransferase
	S. typhi TY2	3275053…3275916 (-)	t3183	Conserved hypothetical protein
	S. typhimurium LT2	3432400…3433263 (-)	STM3263	Putative methyltransferase
	Synechocystis sp. PCC6803	1691493…1692332 (-)	sll0818	Tetrapyrrole methylase family protein
	Y. enterocolictica	4067751…4068650 (-)	YE3726	Hypothetical protein
	Y. pestis KIM**	125918…126817 (-)	y0117	Hypothetical protein
	Y. pseudotuberculosis YPIII	41575324…4158423 (-)	YPTB3492	Possible tetrapyrrole methylase family protein

*http://img.jgi.doe.gov/cgi-bin/pub/main.cgi; **Not observed
