# Supplementary material for: Comparative Bacterial Proteomics: Analysis of the Core Genome Concept
Source: PLoS One. 2008 Feb 6;3(2):e1542. doi: 10.1371/journal.pone.0001542 (PMC2213561; doi:10.1371/journal.pone.0001542)
Supplement: Table S2 — Conserved proteins from different sub-sets of bacteria relative to S. typhimurium. (0.12 MB PDF) [file pone.0001542.s003.pdf]

Table S2. Conserved proteins from different sub-sets of bacteria relative to *S. typhimurium*.

Gene Associated Description	Symbol
***Salmonella* species and *Yersinia* species**	
S. typhimurium: putative outer membrane lipoprotein	YjeL
S. typhimurium: putative cytoplasmic protein	YihD
S. typhimurium: putative hydrolase of the HAD superfamily	YigL
S. typhimurium: putative inner membrane protein	YicH
S. typhimurium: putative outer membrane lipoprotein	YiaF
S. typhimurium: putative periplasmic protein	YhcB
S. typhimurium: putative outer membrane protein	YgiW
S. typhimurium: putative tRNA/rRNA methyltransferase	YfiF
S. typhimurium: putative formate acetyltransferase	YfiD
S. typhimurium: putative transcriptional regulator of two-component regulator protein	YfhG
S. typhimurium: putative cytoplasmic protein	YeeX
S. typhimurium: putative periplasmic protein	YdgH
S. typhimurium: putative cytoplasmic protein	YciN
S. typhimurium: putative lipoprotein	YbjP
S. typhimurium: putative cytoplasmic protein	YbiU
S. typhimurium: putative hydrolase of the alpha/beta superfamily	YafA
S. typhimurium: putative cytoplasmic protein	YaeH
S. typhimurium: modulator of enterobacterial common antigen	WzzE
S. typhimurium: CDP-diacylglycerol phosphotidylhydrolase	UshB
S. typhimurium: vancomycin sensitivity	SanA
S. typhimurium: putative fimbriae usher	SafC
S. typhimurium: putative fimbriae assembly chaparone	SafB
S. typhimurium: RNase II, mRNA degradation	Rnb
S. typhimurium: aspartate carbamoyltransferase, regulatory subunit	PyrL
S. typhimurium: outer membrane protease, receptor for phage OX2	OmpX
S. typhimurium: ABC superfamily	MglB
S. typhimurium: Sugar Specific PTS family, mannose-specific enzyme IIC	ManY
S. typhimurium: Sugar Specific PTS family, mannose-specific enzyme IIAB	ManX
S. typhimurium: periplasmic protein of mal regulon	MalM
S. typhimurium: GMP reductase	GuaC
S. typhimurium: phosphoenolpyruvate-dependent sugar phosphotransferase system, EIIA 2	FruF
S. typhimurium: membrane protein	DamX
S. typhimurium: 6-phospho-beta-glucosidase A	BglA
S. typhimurium: asparagine synthetase A	AsnA
S. typhimurium: ABC superfamily	ArtP
S. typhimurium: putative secreted protein	
S. typhimurium: putative periplasmic transport protein	
S. typhimurium: putative outer membrane lipoprotein	
S. typhimurium: putative inner membrane protein	
S. typhimurium: putative inner membrane lipoprotein	
***Salmonella* species, *Yersinia* species, and *Shewanella oneidensis* MR1**	
S.oneidensis MR-1: activator of ProP osmoprotectant transporter	ProQ
S.oneidensis MR-1: aldehyde-alcohol dehydrogenase	AdhE
S.oneidensis MR-1: bifunctional aspartokinase II/homoserine dehydrogenase, methionine-sensitive	MetL

S.oneidensis MR-1: cell division protein	ZipA
S.oneidensis MR-1: DNA-binding protein, H-NS family	
S.oneidensis MR-1: DUF1439 family lipoprotein complement	
S.oneidensis MR-1: expressed lipoprotein	
S.oneidensis MR-1: expressed lipoprotein	NlpB
S.oneidensis MR-1: expressed protein	
S.oneidensis MR-1: expressed protein complement	
S.oneidensis MR-1: expressed protein of unknown function DUF1260	
S.oneidensis MR-1: expressed protein of unknown function DUF1342	
S.oneidensis MR-1: expressed protein of unknown function DUF1414	
S.oneidensis MR-1: expressed protein of unknown function DUF1451	
S.oneidensis MR-1: FKBP-type peptidyl-prolyl ci-trans isomerase	FkpA
S.oneidensis MR-1: formate acetyltransferase	PlfB
S.oneidensis MR-1: inner membrane sulphatase-like protein complement	
S.oneidensis MR-1: outer membrane MipA family protein	MipA
S.oneidensis MR-1: outer membrane YfaZ beta barrel family protein	YfaZ
S.oneidensis MR-1: PTS system glucose-specific IIBC component	PtsG
S.oneidensis MR-1: pyruvate formate-lyase 1 activating enzyme	PflA
S.oneidensis MR-1: rare lipoprotein B complement	
S.oneidensis MR-1: ribosomal protein L25	RplY
S.oneidensis MR-1: small conductance mechanosensitive ion channel	MscS
S.oneidensis MR-1: subfamily M16A unassigned peptidases	
S.oneidensis MR-1: transcriptional regulator of fatty acid metabolism	FadR

* The name of the organism at the beginning of each description indicates the annotation used to describe each protein.
