# Supplementary material for: Comparative Bacterial Proteomics: Analysis of the Core Genome Concept
Source: PLoS One. 2008 Feb 6;3(2):e1542. doi: 10.1371/journal.pone.0001542 (PMC2213561; doi:10.1371/journal.pone.0001542)
Supplement: Table S3 — Core proteome proteins observed in E. coli K12 MG1655 and S. typhimurium and their published genes noted as essential. (Source: Gerdes, et al. 2003. J. Bacteriol. 185(19):5673–5684; Knuth, et al. 2004. Mol Microbiol. 51(6):1729–1744) (0.25 MB PDF) [file pone.0001542.s004.pdf]

Table S3. Core proteome proteins observed in *E. coli* K12 MG1655 and *S. typhimurium* and their published genes noted as essential. (Source: Gerdes, et al. 2003. *J. Bacteriol.* 185(19):5673-5684; Knuth, et al. 2004. *Mol Microbiol.* 51(6):1729-1744)

Core Protein	*E. coli* Locus Tag	Essential in *E. coli*	*S. typhimurium* Locus Tag	Essential in *S. typhimurium*	Gene Symbol	Description
prot2	NT01EC2079	X	STM1336		*rplT*	Ribosomal protein L20
prot15	NT01EC2458		STM2076		*hisA*	Phosphoribosylformimino-5-aminoimidazole carboxamide ribotide isomerase
prot29	NT01EC3847		STM3304			Ribosomal protein L21 complement
prot30	NT01EC3846		STM3303			Ribosomal protein L27 complement
prot31	NT01EC3844	X	STM3301			GTP-binding protein, GTP1/Obg family complement
prot33	NT01EC0287		STM0322		*proA*	Gamma-glutamyl phosphate reductase
prot50	NT01EC3354	X	STM2953			CTP synthase complement
prot107	NT01EC1332		STM1195	X	*fabG*	3-oxoacyl-(acyl-carrier-protein) reductase
prot128	NT01EC3262		STM2829		*recA*	RecA protein
prot176	NT01EC2082	X	STM1333	X	*thrS*	Threonyl-tRNA synthetase
prot180	NT01EC3043	X	STM2526		*ndk*	Nucleoside diphosphate kinase
prot182	NT01EC0026	X	STM0046	X	*ileS*	Isoleucyl-tRNA synthetase complement
prot185	NT01EC5208	X	STM4475	X	*clpX*	Valyl-tRNA synthetase
prot186	NT01EC0538		STM0449		*tig*	ATP-dependent Clp protease, ATP-binding subunit
prot188	NT01EC0534		STM0447			Trigger factor
prot203	NT01EC1985	X	STM1449	X	*tyrS*	Tyrosyl-tRNA synthetase
prot211	NT01EC3831		STM3290		*argG*	Argininosuccinate synthase
prot221	NT01EC2075		STM1338	X	*pheT*	Phenylalanyl-tRNA synthetase, beta subunit
prot222	NT01EC2076	X	STM1337		*pheS*	Phenylalanyl-tRNA synthetase, alpha subunit
prot326	NT01EC0016		STM0013		*dnaJ*	Chaperone protein
prot330	NT01EC3107		STM2583		*lepA*	GTP-binding protein
prot334	NT01EC0791	X	STM0648		*leuS*	Leucyl-tRNA synthetase
prot338	NT01EC3555	X	STM3090	X	*metK*	S-adenosylmethionine synthetase
prot339	NT01EC4426		STM3730			Bifunctional 4-phosphopantothenoylcysteine decarboxylase/phosphopantothenoylcysteine synthetase
prot341	NT01EC4437	X	STM3740		*gmk*	Guanylate kinase
prot342	NT01EC0036		STM0067		*carB*	Carbamoyl-phosphate synthase, large subunit
prot345	NT01EC5194		STM4460		*pyrB*	Aspartate carbamoyltransferase
prot351	NT01EC2823		STM2384		*aroC*	Chorismate synthase
prot354	NT01EC3260	X	STM2827			Alanyl-tRNA synthetase complement

prot355	NT01EC3982		STM3416		*rpsD*	Ribosomal protein S4
prot361	NT01EC2267	X	STM1901	X	*aspS*	Aspartyl-tRNA synthetase
prot424	NT01EC0113	X	STM0133	X	*ftsZ*	Cell division protein
prot426	NT01EC0109	X	STM0129			UDP-N-acetylmuramate--alanine ligase complement
prot427	NT01EC0108	X	STM0128	X	*murG*	Undecaprenyldiphospho-muramoylpentapeptide beta-N-acetylglucosaminyltransferase
prot429	NT01EC0106	X	STM0126			UDP-N-acetylmuramoylalanine--D-glutamate ligase complement
prot480	NT01EC2459		STM2077		*hisF*	Imidazole glycerol phosphate synthase, cyclase subunit
prot488	NT01EC4095	X	STM3483		*rpe*	Ribulose-phosphate 3-epimerase
prot490	NT01EC3974	X	STM3407		*fmt*	Methionyl-tRNA formyltransferase
prot513	NT01EC3536	X	STM3069		*pgk*	Phosphoglycerate kinase
prot519	NT01EC4946		STM4254		*uvrA*	Excinuclease ABC, A subunit
prot526	NT01EC4688	X	STM3999	X	*polA*	DNA polymerase I
prot560	NT01EC1549	X	STM1714		*topA*	DNA topoisomerase I
prot578	NT01EC4870		STM4175		*purD*	Phosphoribosylamine-glycine ligase
prot698	NT01EC0645	X	STM0537		*cysS*	Cysteinyl-tRNA synthetase
prot714	NT01EC1331	X	STM1194	X	*fabD*	Malonyl CoA-acyl carrier protein transacylase
prot723	NT01EC3151	X	STM2673		*rplS*	Ribosomal protein L19
prot729	NT01EC3156	X	STM2677		*fth*	Signal recognition particle protein
prot749	NT01EC2906	X	STM2415		*gltX*	Glutamyl-tRNA synthetase
prot750	NT01EC4581		STM3903		*llvE*	Branched-chain-amino-acid transaminase
prot752	NT01EC2557	X	STM2155	X	*metG*	Methionyl-tRNA synthetase
prot756	NT01EC4582		STM3904		*llvD*	Dihydroxy-acid dehydratase
prot774	NT01EC3568		STM3103		*rdgB*	Non-canonical purine NTP pyrophosphatase, rdgB/HAM1 family
prot789	NT01EC1466		STM1776		*prfA*	Peptide chain release factor 1, PrfA complement
prot854	NT01EC0116	X	STM0136		*secA*	Preprotein translocase ATPase subunit
prot879	NT01EC4621	X	STM3938		*hemC*	Hydroxymethylbilane synthase
prot901	NT01EC4701	X	STM4009		*bipA*	Virulence regulator BipA complement
prot902	NT01EC1457		STM1784			GTPase/translation factor
prot921	NT01EC3031		STM2510			GMP synthase complement
prot926	NT01EC5065		STM4329		*groES*	Chaperonin (GroES)
prot942	NT01EC3902	X	STM3344		*rpsI*	Ribosomal protein S9
prot943	NT01EC3903	X	STM3345		*rplM*	Ribosomal protein L13
prot953	NT01EC3981	X	STM3415	X	*rpoA*	DNA-directed RNA polymerase, alpha subunit

prot954	NT01EC3983		STM3417		*rpsK*	Ribosomal protein S11
prot955	NT01EC3984		STM3418		*rpsM*	Ribosomal protein S13
prot960	NT01EC3987	X	STM3421	X	*rplO*	Ribosomal protein L15
prot962	NT01EC3990	X	STM3423	X	*rpsE*	Ribosomal protein S5
prot963	NT01EC3991	X	STM3424		*rplR*	Ribosomal protein L18
prot964	NT01EC3993	X	STM3425	X	*rplF*	Ribosomal protein L6
prot965	NT01EC3995	X	STM3426		*rpsH*	Ribosomal protein S8
prot966	NT01EC3997	X	STM3428	X	*rplE*	Ribosomal protein L5
prot967	NT01EC3998	X	STM3429		*rplX*	Ribosomal protein L24
prot968	NT01EC4000	X	STM3430		*rplN*	Ribosomal protein L14
prot971	NT01EC4004	X	STM3433	X	*rplP*	Ribosomal protein L16
prot972	NT01EC4005	X	STM3434		*rpsC*	Ribosomal protein S3
prot973	NT01EC4006	X	STM3435		*rplV*	Ribosomal protein L22
prot975	NT01EC4010	X	STM3437	X	*rplB*	Ribosomal protein L2
prot976	NT01EC4011	X	STM3438		*rplW*	Ribosomal protein L23
prot977	NT01EC4013	X	STM3439	X	*rplD*	Ribosomal protein L4
prot978	NT01EC4015	X	STM3440	X	*rplC*	Ribosomal protein L3
prot982	NT01EC4040	X	STM3447	X	*rpsG*	Ribosomal protein S7
prot983	NT01EC4042	X	STM3448	X	*rpsG*	Ribosomal protein S12
prot987	NT01EC4847	X	STM4152	X	*rplL*	Ribosomal protein L7/L12
prot988	NT01EC4846	X	STM4151		*rplJ*	Ribosomal protein L10
prot990	NT01EC4844		STM4150		*rplA*	Ribosomal protein L1
prot991	NT01EC4842		STM4149		*rplK*	Ribosomal protein L11
prot1105	NT01EC3822	X	STM3282		*pnpA*	Polyribonucleotide nucleotidyltransferase
prot1106	NT01EC3823	X	STM3283		*rpsO*	Ribosomal protein S15
prot1113	NT01EC3826	X	STM3286	X	*infB*	Translation initiation factor IF-2 (InfB)
prot1121	NT01EC3040	X	STM2523		*ispG*	4-hydroxy-3-methylbut-2-en-1-yl diphosphate synthase
prot1132	NT01EC0202	X	STM0219		*frr*	Ribosome recycling factor
prot1135	NT01EC0197	X	STM0216		*rpsB*	Ribosomal protein S2
prot1153	NT01EC2920	X	STM2427	X	*ligA*	DNA ligase, NAD-dependent
prot1170	NT01EC1458	X	STM1783		*pth*	Peptidyl-tRNA hydrolase
prot1172	NT01EC1462	X	STM1780			Ribose-phosphate pyrophosphokinase
prot1200	NT01EC3353		STM2952	X	*eno*	Enolase
prot1215	NT01EC0648		STM0542	X	*folD*	Bifunctional 5,10-methylene-tetrahydrofolate dehydrogenase/5,10-methylene-tetrahydrofolate cyclohydrolase

prot1225	NT01EC4871		STM4176		*purH*	Bifunctional IMP cyclohydrolase/phosphoribosylaminoimidazolecarboxamide formyltransferase
prot1286	NT01EC1088	X	STM0963	X	*serS*	Seryl-tRNA synthetase
prot1315	NT01EC4500		STM3837			DNA polymerase III, beta subunit
prot1333	NT01EC5137	X	STM4394		*rpLL*	Ribosomal protein L9
prot1443	NT01EC2802		STM2362		*purF*	Family C44 non-peptidase homologues
prot1462	NT01EC3160	X	STM2681		*grpE*	Heat shock protein
prot1463	NT01EC0015	X	STM0012	X	*dnaK*	Chaperone protein
prot1493	NT01EC5109		STM4366		*purA*	Adenylosuccinate synthetase
